# β2-Integrins – Regulatory and Executive Bridges in the Signaling Network Controlling Leukocyte Trafficking and Migration

**DOI:** 10.3389/fimmu.2022.809590

**Published:** 2022-04-22

**Authors:** Carla Guenther

**Affiliations:** ^1^ Department of Veterinary Biosciences, University of Helsinki, Helsinki, Finland; ^2^ Department of Molecular Immunology, Research Institute for Microbial Diseases, Osaka University, Osaka, Japan; ^3^ Laboratory of Molecular Immunology, Immunology Frontier Research Center, Osaka University, Osaka, Japan

**Keywords:** leukocyte migration, β2-integrins, RhoGTPases, epigenetics, actin tread milling, amoeboid migration, LFA-1

## Abstract

Leukocyte trafficking is an essential process of immunity, occurring as leukocytes travel within the bloodstream and as leukocyte migration within tissues. While it is now established that leukocytes can utilize the mesenchymal migration mode or amoeboid migration mode, differences in the migratory behavior of leukocyte subclasses and how these are realized on a molecular level in each subclass is not fully understood. To outline these differences, first migration modes and their dependence on parameters of the extracellular environments will be explained, as well as the intracellular molecular machinery that powers migration in general. Extracellular parameters are detected by adhesion receptors such as integrins. β2-integrins are surface receptors exclusively expressed on leukocytes and are essential for leukocytes exiting the bloodstream, as well as in mesenchymal migration modes, however, integrins are dispensable for the amoeboid migration mode. Additionally, the balance of different RhoGTPases – which are downstream of surface receptor signaling, including integrins – mediate formation of membrane structures as well as actin dynamics. Individual leukocyte subpopulations have been shown to express distinct RhoGTPase profiles along with their differences in migration behavior, which will be outlined. Emerging aspects of leukocyte migration include signal transduction from integrins *via* actin to the nucleus that regulates DNA status, gene expression profiles and ultimately leukocyte migratory phenotypes, as well as altered leukocyte migration in tumors, which will be touched upon.

## Introduction

Leukocyte trafficking is a multifaceted process that not only encompasses the cell adhesion cascade, transmigration and migration through tissues, but also gains further depth by the fact that leukocytes can use the mesenchymal migration mode as well as a unique amoeboid migration mode that sets them apart from other cell types. Additionally, our understanding of leukocyte migration is rapidly evolving to the extent that not differentiating between these aspects – be it transmigration, crawling on 2D or migration through 3D environments – and instead refering to them in equal fashion as ‘leukocyte migration’ hinders us from appreciating the true complexity of these processes. The importance of this is heightened by the fact that leukocyte subpopulations show distinct migratory behavior across all these aspects. Additionally, while it is now appreciated that environmental parameters dictate which migration mode is possible for all leukocytes and these parameters are integrated into the cell by adhesion receptors such as β2-integrins, it is becoming clear that how the appropriate migration modes are executed is down to a distinct combination of β2-integrin downstream components which are, in turn, acting uniquely and are expressed in different leukocyte populations. It is clear that our collective understanding of leukocyte migration is just scratching the surface and this is due to several factors. One hindering factor is that not all aspects of leukocyte migration are researched to the same degree. Transwell migration assays represent the vast majority of assays used to study leukocyte migration; this means we have much more data on transmigration than on migration in tissues. Additionally, the 2D or 3D migration assays which are carried out often rely on single-protein coatings or matrices, respectively, and this fails to represent the complexity of living endothelium and tissues. Furthermore, the structural organization of proteins in bundles or fibers is neglected, just as is their stiffness and elasticity. Finally, leukocyte subpopulations such as neutrophils, macrophages and T-cells have been studied most, while eosinophils and basophils have been almost entirely ignored. This review seeks to outline some of the field’s complexity and why the aforementioned aspects of the environment are important, in the context of β2-integrins, which are essential for the leukocyte adhesion cascade.

## The Leukocyte Adhesion Cascade

**Figure 1:**
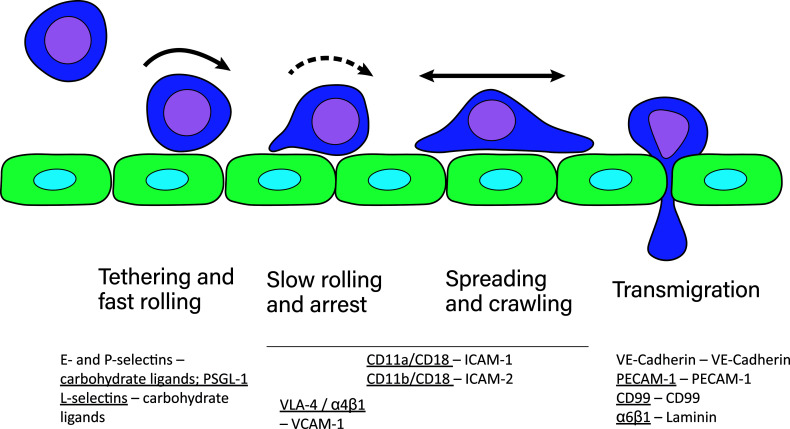
The cell adhesion cascade. During adhesion to and extravasation through the endothelium, leukocytes undergo several steps, namely fast rolling and tethering, slow rolling and arrest, spreading and crawling and lastly transmigration. These steps are mediated by several different adhesion receptors expressed by leukocytes, indicated by underscore, and the endothelium (2). P-selectins and E-selectins expressed by the endothelium bind to their carbohydrate ligands on leukocytes during fast rolling. Leukocytes express L-selectins, which bind to ligands on the endothelium during fast rolling. The most well known selectin ligand is P-selectin glycoprotein ligand-1 (PSGL-1). Selectin-ligand binding results in integrin activation in leukocytes, which then mediates slow rolling, arrest, spreading and crawling. During transmigration, several homophilic adhesion receptors, including VE-cadherins important for tight junctions between endothelial cells, and CD99 and PECAM-1 are expressed by endothelial cells and leukocytes. Additionally, leukocytes express α6β1 integrins that bind to laminin.

The majority of leukocyte trafficking occurs in the bloodstream, in which leukocytes – typically T- and B-cells, neutrophils, monocytes, eosinophils and basophils – are passively carried by the bloodstream close to sites of infections. In response to infections, the endothelium lining the blood vessels closest to the site of infection expresses chemokines and adhesion receptor ligands which initiate and mediate the cell adhesion cascade. This cascade is the process through which leukocytes leave the bloodstream and extravasate into the tissue. In this multistep process, leukocytes first tether on the endothelium, followed by fast and slow rolling along the vessel wall until the leukocyte is arrested on the endothelium (1, 2). Leukocytes then typically begin to spread and crawl across the endothelium (3). The distinctive steps in the adhesion cascade are: step 1 – tethering and fast rolling, step 2 – slow rolling and arrest, step 3 – spreading and crawling, step 4 – transmigration. The adhesion cascade step of crawling across the endothelium has been termed locomotion (3). This process is depicted in **Figure 1** and has been reviewed multiple times in detail [for example (4)]. Selectins are the first adhesion receptors mediating tethering and fast rolling during which their activity typically overlaps with β2-integrins (4). β2-integrins are especially crucial for the fast and slow rolling, cell arrest, and spreading and crawling steps (4). They have been identified as mechanoreceptors (5) and therefore likely play a role in probing the endothelium for points of exit as well. Further detail in regards to involvement of each adhesion receptor at each step and their typical ligands are depicted and described in **Figure 1**.

**Figure 2:**
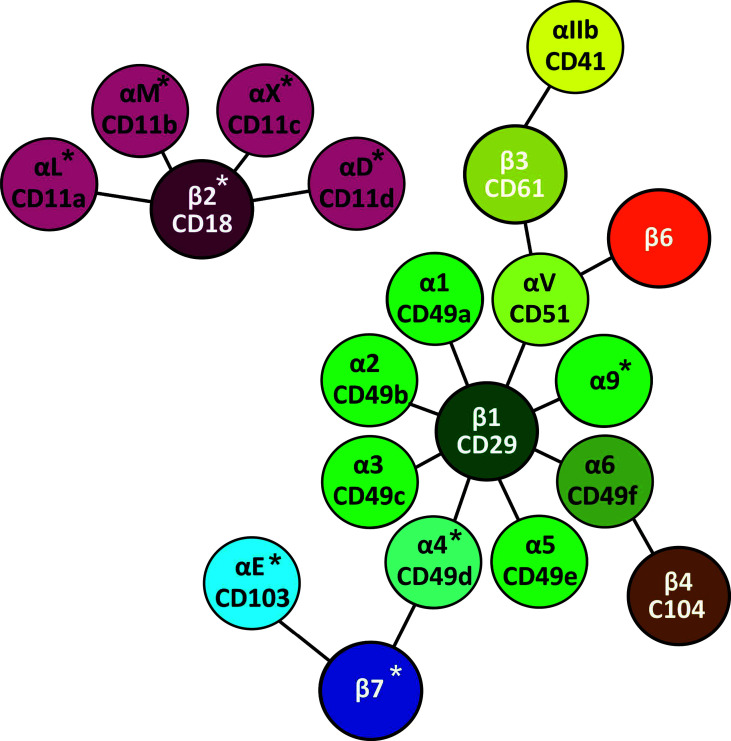
Leukocyte integrins. Leukocyte integrins arranged around their β chains. α and β chains marked with * are specific for leukocytes (27), while platelets also express β2-integrins (28).

β2-integrins are a group of essential adhesion receptors, consisting of CD11a/CD18 (LFA-1, αLβ2), CD11b/CD18 (MAC1, αMβ2), CD11c/CD18 (αXβ2), and CD11d/CD18 (αDβ2) (6). Leukocytes express several different types of integrins (7) (**Table 1** and **Figure 2**), and they express β2- and β7-integrins as the only cells [β2-integrins are also expressed on platelets (28)]. Furthermore, in most circulating leukocytes β2-integrins are the integrin class expressed in the highest amounts (13). β2-integrins are differently expressed on different leukocytes and they differ in terms of ligand binding. CD11a and CD11d bind limited ligands (CD11a/ICAMs, CD11d/ICAM-3, VCAM-1), while CD11c and CD11b form diverse receptor–ligand interactions with more than 40 identified ligands. β2-integrins have been shown to be essential for pro- as well as anti-inflammatory processes (19, 29–31).

**Table 1:** Leukocyte Integrins And Their Ligands, Expanded On From (8).

Leukocyte	Integrin name	Ligands (most important)
eosinophils	CD11a/CD18 (αLβ2)	ICAM-1, -2, -3, -5
	CD11b/CD18 (αMβ2) (9)	iC3b (11), fibrinogen, heparin, many others (40+)
	CD11d/CD18 (αDβ2)	ICAM-3, VCAM-1
	α4β1 (VLA-4)	VCAM-1, fibronectin
	α6β1 (10)	Laminin
	α4β7 (10)	MAdCAM-1, fibronectin
basophils	CD11a/CD18 (αLβ2)	ICAM-1, -2, -3, -5
	CD11b/CD18 (αMβ2) (9)	iC3b (11), fibrinogen, heparin, many others (40+)
	CD11d/CD18 (αDβ2)	ICAM-3, VCAM-1
	β1 (12)	
	α4β1 (12)	VCAM-1, fibronectin
	α5β1 (12)	Fibronectin
	β3 (12)	
	β4 (12)	
neutrophils	CD11a/CD18 (αLβ2)	ICAM-1, -2, -3, -5
	CD11b/CD18 (αMβ2)	iC3b (11), fibrinogen, heparin, many others (40+)
	CD11d/CD18 (αDβ2)	ICAM-3, VCAM-1
	α2β1 (induced expression) (13)	Collagen, laminin (13)
	α3β1 (induced expression) (13)	Collagen, laminin, fibronectin, tenascin (13)/RGD
	α4β1 (induced expression) (13)	VCAM-1, fibronectin, expression decreases during maturation (14)
	α5β1 (induced expression) (13)	Fibronectin
	α6β1 (induced expression) (13)	Laminin
	α9β1 (induced expression) (13)	VCAM-1, tenascin
	αVβ3 (13)	Arg-Gly-Asp (RGD) (15)
monocytes	CD11a/CD18 (αLβ2)	ICAM-1, -2, -3, -5
	CD11b/CD18 (αMβ2)	iC3b (11), fibrinogen, heparin, many others (40+)
	CD11c/CD18 (αXβ2)	iC3b (11), fibrinogen, heparin, many others
	CD11d/CD18 (αDβ2)	ICAM-3, VCAM-1 (16)
	α4β1	VCAM-1, fibronectin
	α4β7	MAdCAM-1, fibronectin
	α1β1	Collagen
	α2β1	Collagen
	α5β1	Fibronectin
	α6β1	Laminin
macrophages	CD11a/CD18 (αLβ2)	ICAM-1, -2, -3, -5
	CD11b/CD18 (αMβ2)	iC3b (11), fibrinogen, heparin, many others (40+)
	CD11c/CD18 (αXβ2)	iC3b (11), fibrinogen, heparin, many others
	CD11d/CD18 (αDβ2)	ICAM-3, VCAM-1 (16)
	αVβ3 (7)	RGD (15)
dendritic cells	CD11a/CD18 (αLβ2)	ICAM-1, -2, -3, -5
	CD11b/CD18 (αMβ2)	iC3b (11), fibrinogen, heparin, many others (40+)
	CD11c/CD18 (αXβ2)	iC3b (11), fibrinogen, heparin, many others
	CD11d/CD18 (αDβ2) (17)	ICAM-3, VCAM-1 (16)
	α4β1 (18) *	VCAM-1, fibronectin
	α5β1 (18) *	Fibronectin
	αVβ1 (18) *	Vitronectin (15)
	αVβ3 (18) *	RGD (15)
	α4β7 (18) *	MAdCAM-1, fibronectin
	*subunit expression *via* FC	
lymphocytes (general)	CD11a/CD18 (αLβ2)	ICAM-1, -2, -3, -5
	α4β1	VCAM-1, fibronectin
	α4β7	MAdCAM-1, fibronectin
natural killer cells	CD11a/CD18 (αLβ2)	ICAM-1, -2, -3, -5
	CD11b/CD18 (αMβ2)	iC3b (11), fibrinogen, heparin, many others (40+)
	CD11c/CD18 (αXβ2)	iC3b (11), fibrinogen, heparin, many others
	α4β7	MAdCAM-1, fibronectin
	α4β1	VCAM-1, fibronectin
B-cells	CD11a/CD18 (αLβ2)	ICAM-1, -2, -3, -5
	α4β1	VCAM-1, fibronectin
	α4β7	MAdCAM-1, fibronectin
	α1β1	Collagen
	α2β1	Collagen
T-cells	CD11a/CD18 (αLβ2)	ICAM-1, -2, -3, -5
	α4β1	VCAM-1, fibronectin
	α4β7	MAdCAM-1, fibronectin
	α5β1	Fibronectin
	α6β1	Laminin
small fraction circulating T-cells	CD11a/CD18 (αLβ2)	ICAM-1, -2, -3, -5
	CD11d/CD18 (αDβ2)	ICAM-3, VCAM-1 (16)
	α4β1	VCAM-1, fibronectin
	α4β7	MAdCAM-1, fibronectin
	α5β1	Fibronectin
	α6β1	Laminin
intra-epithelial T-cells	CD11a/CD18 (αLβ2)	ICAM-1, -2, -3, -5
	α4β1	VCAM-1, fibronectin
	α4β7	MAdCAM-1, fibronectin
	αEβ7	E-cadherin
long-term activated T-cells	CD11a/CD18 (αLβ2)	ICAM-1, -2, -3, -5
	α4β1	VCAM-1, fibronectin
	α4β7	MAdCAM-1, fibronectin
	α1β1	Collagen
	α2β1	Collagen
mast cells	CD11a/CD18 (αLβ2)	ICAM-1, -2, -3, -5
	CD11b/CD18 (αMβ2) (19)	iC3b (11), fibrinogen, heparin, many others (40+)
	α4β1 (20)	VCAM-1, fibronectin
	α4β7 (21)	MAdCAM-1, fibronectin
	αVβ6 (22)	tenascin, fibronectin (23)
	α2β3 (24)	fibrinogen
microglia	CD11a/CD18 (αLβ2)	ICAM-1, -2, -3, -5
	CD11b/CD18 (αMβ2) (25)	iC3b (11), fibrinogen, heparin, many others (40+)
	CD11c * (26)	iC3b (11), fibrinogen, heparin, many others
	CD11d * (26)	ICAM-3, VCAM-1 (273)
	α1 subunit * (26)	
	α2 subunit * (26)	
	α2b subunit * (26)	
	α3 subunit * (26)	
	α5 subunit * (26)	
	α6 subunit * (26)	
	α7 subunit * (26)	
	α8 subunit * (26)	
	α9 subunit * (26)	
	α11 subunit * (26)	
	β1 subunit * (26)	
	β3 subunit * (26)	
	β4 subunit * (26)	
	β5 subunit * (26)	
	β7 subunit * (26)	
	β8 subunit * (26)	
	*subunit expression *via* RNA-sequencing, positive glia-brain_log fold change	

Integrins expressed by leukocytes and their ligands sorted by leukocyte type.

**Figure 3:**
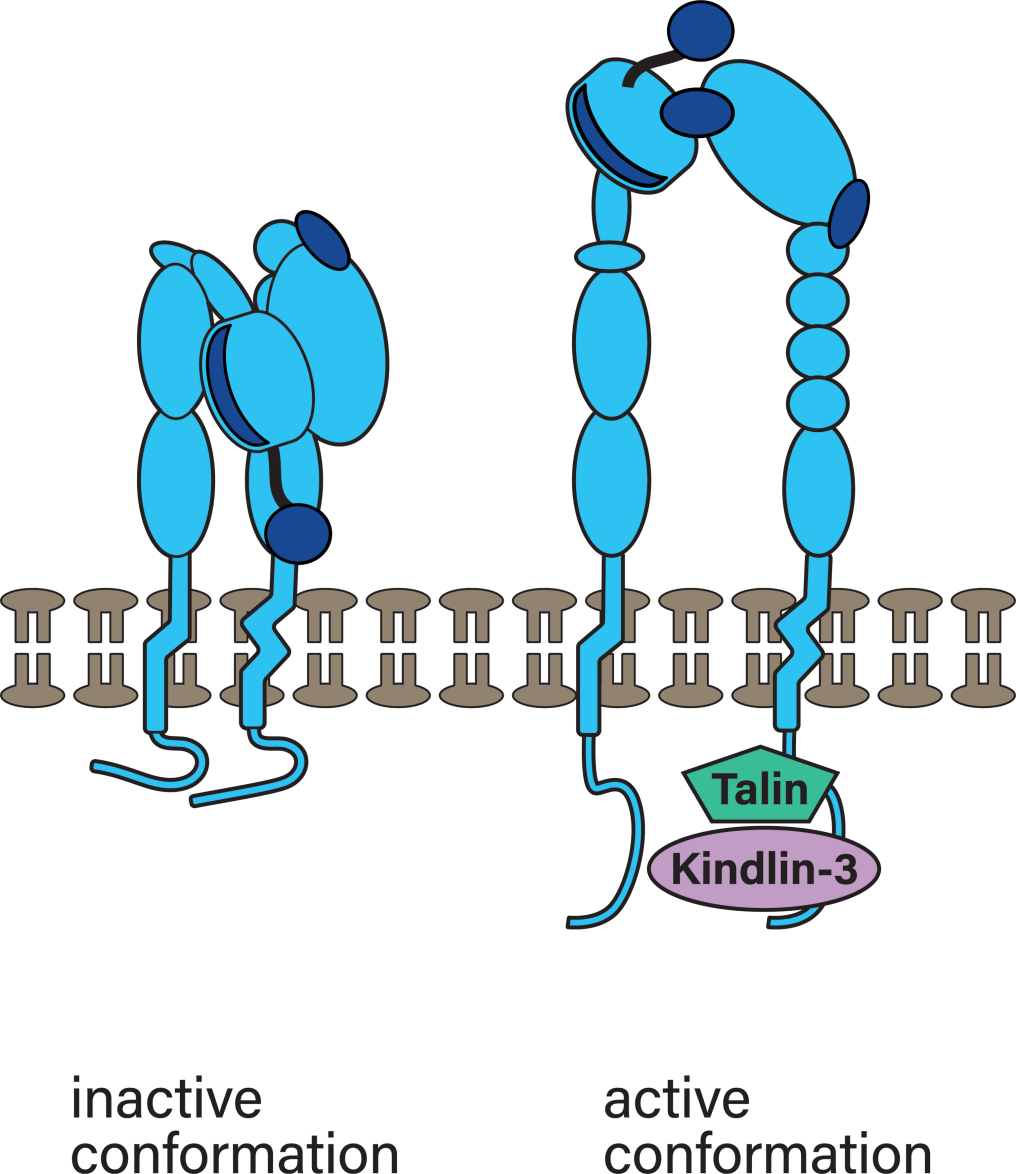
Integrin conformation. In leukocytes traveling through the bloodstream, in absence of ligands, 90% of integrins are in the bent inactive conformation, depicted on the left. Inside-out signaling as a result of chemokine receptor/ligand and selectin/ligand binding during fast rolling results in talin and kindlin-3 binding to β chains which results in unbending and changing to the active conformation (right). The unbending results in ligand binding sites being revealed.

Integrins can signal from the inside to the outside, which is especially important during the cell adhesion cascade. A central aspect of such signaling is typically conformational changes of integrins, which in turn regulate the individual bond strength between individual integrin heterodimers and ligands (30, 32). Integrins can only bind ligands if they are in an active – meaning open, extended (high affinity) conformation (33) (depicted in **Figure 3**). A ‘bent’, inactive integrin conformation (33) predominates when leukocytes travel through the bloodstream to prevent cell aggregation (19). For example, a 9:1 ratio between inactive and active conformations was established for a mutant form of CD11b in the absence of ligands (34, 35). When leukocytes bump into endothelial cells producing chemokines and expressing selectin ligands, chemokine-receptor and selectin engagement initiates the binding of talin and kindlin to integrin cytoplasmic tails (36, 37). This leads to integrin switching from the bent into the extended conformation, akin to a switch blade, and is an example of integrin inside-out signaling. This conformational switch reveals ligand binding sites in the extracellular domain in parts that were facing the cell membrane in the bent conformation (38). At the same time, the conformational switch leads to integrin cytoplasmic tail separation which enables the binding of further interaction partners like tensin and vinculin (39).

## Leukocyte Migration

### Transmigration

During crawling and spreading, leukocytes probe the endothelium for suitable sites of exit, through which they transmigrate. Such sites of exit can be cell–cell junctions, but they also travel through endothelial cell bodies (1, 4, 40, 41). Monocytes have been shown to typically choose gap-junctions to transmigrate. If crawling/locomotion is disabled, for example due to CD11b/CD18 knockdown, neutrophils transmigrate transcellularly, meaning through endothelial cells (3). During transmigration, leukocytes change their integrin expression profiles, for example α6β1-integrin is upregulated, which mediates transmigration (4). Transmigration is the migration process modeled best in transwell/Boyden chamber assays (42). This assay consists of two champers, one filled with normal media and one with media including chemokines. These chambers are separated by a porous membrane, which creates a chemokine gradient (43). However, these assays fail to present leukocyte–endothelial cell interactions as well as gap junctions. Based on the number of papers tagged with ‘transwell leukocyte migration’ vs ‘2D leukocyte migration’ or ‘3D leukocyte migration’ in Pubmed to date, transwell assays are the most used assay to investigate leukocyte migration, however, data generated with this model is often referred to as ‘migration’ or ‘chemotaxis’ instead of ‘transmigration’.

### Leukocytes Navigate Different Microenvironments During Migration

After transmigrating, leukocytes proceed to actively migrate through tissues towards the site of infection, guided by chemokines. This means all migrating leukocytes navigate numerous tissues that present different microenvironments and necessitate different migration modes. This is especially true for leukocytes that migrate long distances. Leukocytes, that have transmigrated from the bloodstream, however typically migrate relatively short distances. For example, the typical maximum distance of (tissue) cells to their nearest blood vessel is 100 µm and rarely exceeds 200 µm (44), and leukocytes likely don’t migrate into other tissues or microenvironments. Leukocytes that actively migrate long distances across several microenvironments are typically antigen–presenting cells, such as dendritic cells, neutrophils and macrophages (45, 46), which migrate through the tissues towards and transmigrate into the lymph system. The lymph system is closely related to and connected to the blood system, however, lymph flow rates are much lower. For example, in mice the blood flow rate in microvessels (45.8 µm diameter) in the retina can be up to 1.55 µl/min (47), while in the lymph system a flow rate of 0.3 µl/h has been measured (48). The slow flow rates necessitate the active migration of leukocytes to the nearest draining lymph node to present antigens. Besides antigen presenting cells, high numbers of T-cells and low numbers of monocytes, all types of granulocytes, and B-cells have also been found in afferent lymph vessels (49–53), however, it is unclear how much active migration is involved in the presence of these leukocytes. Additionally, mast cells migrate from the skin to lymph nodes after UV exposure (54).

The most obvious and most acknowledged difference in the microenvironments that leukocytes migrate through is dimensionality. Crawling on the endothelium of blood- or lymph vessels happens in 2D environments (4, 55) and migration through tissues happens in 3D environments (56). While direct *in vivo* microscopy has shed much light on migratory processes (57, 58), the molecular mechanisms are often investigated using *in vitro* models. 2D migration is often modeled in assays using Zigmond or Dunn chambers, which involves two or three tissue plastic chambers, one or two of which are filled with media containing chemokines and one without chemokines to establish a chemokine gradient (59). Since 2D migration *in vivo* typically happens during crawling on the endothelium (4, 55), leukocyte–endothelial cell interactions are neglected in these models as are shear forces from the bloodstream.

3D migration is typically investigated using collagen or matrigel matrices in which cells are embedded (60). These matrices can be sandwiched between chambers of media with different initial chemokine concentrations, establishing a chemokine gradient. One example of a commercially-available setup is ‘µ-Slides Chemotaxis’ by ibidi (59). Additionally gel matrices can be set atop transwells to first model 3D migration and then transmigration (61), however, direct observation of 3D migration would be difficult. The downside of collagen and matrigel matrices is that they fail to replicate the complexity of tissues, be it in structure or in the variety of components. Cancer spheroids are used to model leukocyte invasion into tumors (62). These are often limited to cancer cells and fail to account for the stroma that support cancer progression. *Ex vivo* models are also used, in which mice ears are utilized that allow direct observation by microscopy of fluorescently labeled leukocytes (63). Mice ears offer few specific tissue environments for leukocytes to migrate in and would not be comparable to other tissues and organs.

### Leukocyte Migration Modes

On 2D surfaces such as the endothelium of blood and lymph vessels (55), leukocytes use the mesenchymal migration mode, which is heavily dependent on integrins. Monocyte crawling on the endothelium of blood vessels is particularly dependent on CD11b/CD18 and ligand ICAM-1 (3, 64).

The molecular mechanism of the mesenchymal migration mode is the following: Integrins establish adhesion sites at the edge of the cell in the direction the cell is migrating. At this leading edge, lamellipodia are formed, that contain adhesion receptors, which bind ligands and transduce pulling forces (65). The cell then pulls its body towards this adhesion site. Meanwhile, at the cell rear the adhesion sites are broken and recycled to the front. The force required for the cell’s movement is generated by the actin filament treadmill. This treadmill consists of actin polymerization at the leading cell edge and actin depolymerization at the cell rear (66–68). Actin polymerization ultimately generates power by pushing against elastic proteins that mediate the force by unbending at the cell front (69). The actin network as a whole presses against the cell’s leading edge and the membrane that forms the lamellipodia containing the adhesion receptors (65).

Leukocytes migrate differently in 3D. In particular, dendritic cells (during homeostasis) (18, 53) and neutrophils (70) have been shown to use an amoeboid migration mode independent of integrins. So instead of the actin treadmill, this mode is facilitated by actin polymerization and squeezing and pushing (18). In a groundbreaking study, Lämmermann et al. knocked down all integrins in dendritic cells and proved that this migration mode is independent of integrin-mediated adhesion (18). Instead of lamellipodia, such migrating cells form blebs that do not strongly adhere or pull on the substrate (18, 71–73).

**Figure 4:**
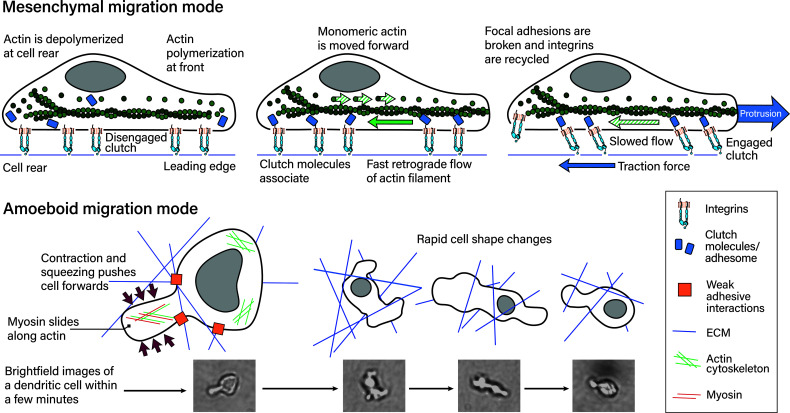
Mesenchymal and amoeboid migration modes. The mesenchymal migration mode, depicted on top, is mediated by strong focal adhesions including integrin–ligand bonds to the substrate. Actin polymerization at the cell’s leading edge and actin retrograde flow powers this migration mode, which requires the counterforce of the adhesion sites. In more detail (77): (left) Actin is polymerized at the leading edge while it is depolymerized at the rear. In this depiction adhesome/clutch proteins have not bound integrin cytoplasmic tails and are instead distributed in the cytoplasm. This means the integrin-actin clutch is disengaged. (middle) Actin polymerization at the front necessitates actin monomers being moved to the front. The growing actin polymer at the front pushes against the elastic fibers at the cell membrane and generates an actin retrograde flow relative to actin’s position in the cell. If the integrin-actin clutch is not engaged this flow is fast. Integrin–ligand bonds are reinforced by adhesome protein binding, which results in focal adhesions, along with the metaphorical clutch, being formed. The clutch is still disengaged if the clutch does not bind actin. (right) The adhesome proteins within the clutch mediate binding to polymerized actin moving towards the rear. This results in slowing of the retrograde actin flow, traction force generation and ultimately protrusion. At the cell rear, focal adhesions are broken and proteins, including integrins recycled. During amoeboid migration (bottom), leukocytes exert weak adhesive forces on the extracellular matrix. This mode is powered by myosin II sliding along actin filaments at the cell’s rear, which translates into contraction and squeezing forces that push the cytoplasm and cell body forwards. Leukocytes find their paths through complex 3D environments and navigating these involves rapid cell shape changes within minutes and shorter, as depicted on the right. The reference pictures were taken during a timelapse 3D dendritic cell migration experiment through a collagen matrix and are of a single dendritic cell, with most cell shapes not being depicted.

Mechanistically, this migration mode is powered by myosin II filaments sliding along actin filaments and thereby generating contractile forces (65). These contractile forces are present in the cell’s rear and push the cytoplasmic fluid (74), as well as move the cell body (71) forward. At a molecular level, actin and myosin II form the actomyosin complex. In this complex, myosin is bound and travels along actin filaments in an ATP-dependent manner. Myosin travels by binding to consecutive actin binding sites further along the actin filament and in this manner generates force (75, 76). Consecutive binding and detachment in turn is dependent on conformational changes that induce rotation of myosin’s motor protein structures that lead to the ‘power stroke’. The power stroke signifies myosin changing from one actin binding site to another (75). Both migration modes are depicted in **Figure 4**.

The regulation of the two different cell migration modes occurs by a combination of both intracellular mechanisms and sensed extracellular parameters. Intracellularly, cell migration modes are regulated by cell shape and adhesiveness. These processes in turn are regulated by the balance of Ras homologous A (RhoA), Ras-related C3 botulinum toxin substrate (Rac) proteins and cell division cycle 42 (Cdc42), which are small GTPases central to actin remodeling dynamics and are characterized further below.

**Figure 5:**
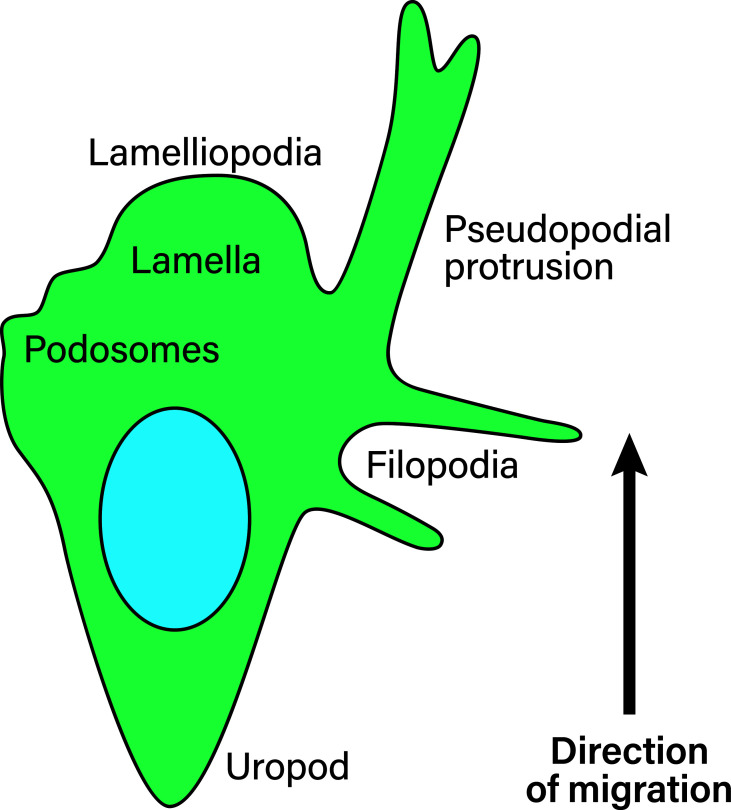
Membrane structures in migrating leukocytes. Actively migrating leukocytes on 2D surfaces form distinct membrane structures. Depicted is the lamellipodia, the actin-rich leading cell edge, the lamella behind it (containing myosin II), as well as the uropod or the cell rear of migrating leukocytes, which are membrane structures that need to be formed during polarization to achieve directional migration. Pseudopodial protrusions are thick, arm-like membrane protrusions in the direction of migration that mediate material uptake. Podosomes are small membrane protrusions which are associated with ECM degradation using proteases. Finally, filopodia are thin finger-like membrane protrusions used to probe the surrounding environment. During amoeboid migration in 3D, leukocytes form small membrane blebs to enact low adhesive forces on the ECM.

Extracellular parameters are sensed by integrins. In order to function properly, integrins are typically located at the edges of filopodia and lamellipodia, which are organized membrane structures that T-cells have been shown to use to navigate the extracellular matrix (ECM) (78), a mechanism most likely shared by all leukocytes (depicted in **Figure 5**). Filopodia are highly dynamic, finger-like protrusions containing parallel bundles of filamentous actin (F-actin) (79). The lamellipodia is a flat membrane protrusion at the leading cell edge, mainly consisting of F-actin, and the persistence of the lamellipodia is important for directionality during cell migration (80). Lamellipodia formation is associated with the mesenchymal migration mode (81). The area directly behind the lamellipodia is termed the lamella and is also relatively flat but contains myosin II (82–84). The lamellipodia has been proposed to be dispensable for cell migration, while the lamella is essential in epithelial cell migration (84). The cell rear of migrating cells is called the uropod. Migrating leukocytes form pseudopodial protrusions (membrane structures that facilitate material uptake) at the leading edge during amoeboid migration (85). Macrophages, dendritic cells and neutrophils form podosomes, which are actin-rich membrane protrusions used to degrade the ECM (86). Additionally, leukocytes form membrane blebs during amoeboid migration modes through which they push into the ECM and generate anchoring stresses, as well as shear stress at bleb protrusions (81). Amoeboid migrating neutrophils in a zebrafish model have been found to form knobs, as the beginning of long trailing extensions that undergo dynamic retractions at the cell rear (87). However, it is not clear what kind of proteins are present in these trailing extensions and what function they serve.

Extracellular matrix parameters that regulate migratory modes include ligand availability (for adhesion receptors), density, stiffness, elasticity and pore size. Of these parameters, pore size is a critical factor. Pores that are many times larger than cells render matrices into essentially 2D surfaces and thereby prevent leukocytes from utilizing the 3D migration mode. Pores that are many times smaller than cells block cell migration (71, 88). The nucleus is a major obstacle in leukocyte migration because it is the stiffest and largest organelle (89, 90), and if a cell is unable to move its nucleus through a pore, it has to find a way around (91–93). In order to efficiently navigate ECM environments, leukocytes use their nuclei as mechanical force sensors to scan their environment for adequate pore sizes (94). Leukocytes can further use their nuclei to push ECM components such as collagen apart, and test ECM qualities like stiffness and elasticity, which sets them apart from other migrating cells, that typically drag their nuclei behind (71, 88, 95, 96). Stiffness has been shown to guide cell migration towards stiffer matrices and this process is called durotaxis (71, 88, 95). Elasticity is closely linked to stiffness. Adhesion receptor availability dictates which migration mode can be used. Integrin ligand availability is critical for the mesenchymal migration mode, which is powered by pushing filaments, causing the membrane to protrude (97). This can only happen if there is actin retrograde flow and this in turn is dependent on a counter force such as integrin engagement (77, 97). Dependent on integrin or adhesion receptor ligand availability, dendritic cells switch from adhesion-dependent to independent modes (97). This is probably some of the most direct evidence regarding how integrins regulate migration modes as engagement of the integrin–actin clutch translates to protrusion, whereas disengagement of this clutch leads to fast actin retrograde flow and slippage (amoeboid migration mode) (77, 97). Basically, ECM parameters dictate which migration modes are possible for leukocytes, while RhoA, Rac and Cdc42 mediate migration modes intracellularly.

## β2-Integrin Signaling

Integrins function as regulatory and executive bridges between these two components since they bind ECM components and are upstream of RhoA and Rac. The function as executive bridges is based on the characteristic integrin signaling in which they ‘integrate’ extracellular information into the inside of the cell. This type of signaling is called ‘outside-in’ signaling. The ability of integrins to mediate ‘outside-in’ signaling is due to their structure as well as binding of a multitude of cytoplasmic interaction partners. Integrins are transmembrane proteins and are linked to the intracellular cytoskeleton *via* interaction partners such as talin and kindlin (98, 99). Extracellular information is further transmitted *via* the actin cytoskeleton directly to the nucleus (100, 101) or is translated into chemical information in a multitude of signaling pathways downstream of integrins. Many such pathways contain focal adhesion kinase (FAK), Src kinase family members, adaptor molecules bearing immunoreceptor tyrosine-based activation motifs (ITAMs, e.g. Fc receptor γ-chain IIa), Syk and Rho GTPases that regulate actin polymerization (98, 102–104). For example, β1-integrins (105, 106) and β2-integrins (17) regulate actin dynamics *via* RhoA. Whether β2-integrin signaling stimulates or inhibits Rac seems to be cell type–dependent. In neutrophils, β2-integrin–mediated adhesion has been shown to downregulate Rac and stimulate Cdc42 (107). In human T-cells, CD11a/CD18 has been shown to activate Rac-1 and Cdc42, which mediates cell adhesion (108).

Inside-out and outside-in signaling can happen seamlessly during adhesion processes. An example is, again, adhesion in the bloodstream. In order to withstand the shear forces leukocytes are subjected to, adhesion sites are strengthened under force. Single adhesion points consisting of individual or a few integrin-ligand bonds can be reinforced by recruiting other integrins to form multiprotein clusters (focal adhesion points). This reinforcement is a result of outside-in signaling followed by inside-out signaling (109).

As indicated throughout, both ways of integrin signaling are mediated by cytoplasmic interaction partners. Cytoplasmic interaction partners and proteins that are recruited to integrin adhesion sites are collectively termed the integrin adhesome. Hundreds to thousands (110) of integrin adhesome proteins have been identified (111, 112); 60 integrin adhesome proteins are shared between integrin classes such as α-actinin, talin, tensin and filamin (113). Talin, kindlin(-2) and filamin crosslink integrins to the actin cytoskeleton (114, 115) and recruit other proteins to mediate downstream signaling *via* Src kinases (Lck, Lyn, Fyn) and Syk (116).

## β2-Integrin Interaction Partners That Link β2-Integrins and RhoGTPases

In the following sections β2-integrin downstream signaling components will be characterized to outline rough pathways before the components’ involvement in migration of individual leukocyte subpopulations is explored.

### Talin

Talin is an important integrin cofactor in general, and its binding to integrin cytoplasmic tails is involved in activation of β3-integrins during inside-out signaling (117). Talin is mechanosensitive and unfolds under applied forces of 5–25 pN (118, 119). In neurons, talin signaling initiates RhoA/ROCK activation *via* Src (120). Talin binds DLC1 (121), which activates RhoA, RhoB, RhoC and Cdc42 and is a negative regulator of RhoA in non-small cell lung carcinoma cells (122).

### Kindlin-3

Kindlin-3 is important for integrin inside-out signaling mediating the active integrin conformation and is essential for proper function of multiple integrin classes (β1, β2 and β3 at least) (123–125). In fact, this signaling is so important that mutations affecting the kindlin-3 gene result in leukocyte adhesion deficiency III (LAD III; mutations mainly affecting CD18 lead to LAD I). LAD III is prevented by the expression of only 5% of the typical kindlin-3 level during homeostasis, however, higher amounts are needed for leukocytes to function properly (126). Kindlin-3 is mainly expressed in the haematopoietic system, so it is important for leukocytes and platelets (123, 127). Kindlin-3 is essential for CD11a/CD18 inside-out signaling (125).

### Filamin A

Filamin A is an integrin cytoplasmic cofactor interacting with β-1, β-2, β-3 and β-7 integrins (128–134), mechanosensitive (135) and an actin crosslinking protein (136). Filamin A-actin crosslinking establishes leading-edge actin networks which distribute forces over the entire cytoskeleton (137). This force distribution is especially important during adhesion to and crawling across the endothelium since it allows cells to withstand shear forces (138).

Stretching Filamin A/actin crosslinks under forces of 2–5 pN (135) leads to Filamin A unfolding and dissociates the FilaminA-binding GTPase-activating protein (FilGAP) from filamin. Instead, Filamin A binding to integrins is increased (139). FilGAP inactivates Rac1 in a Rho-associated kinase (ROCK)-dependent manner, and it inactivates Cdc42 to a lesser extent. The forced expression of Filamin A together with FilGAP decreases active Rac1 amounts, and since FilGAP is localized at lamellae, it inhibits lamellae formation and integrin-mediated cell spreading, which could be important for amoeboid migration (140). Filamin A itself is an important regulator of leukocyte adhesion, migration and homing.

### RhoGTPases

**Figure 6:**
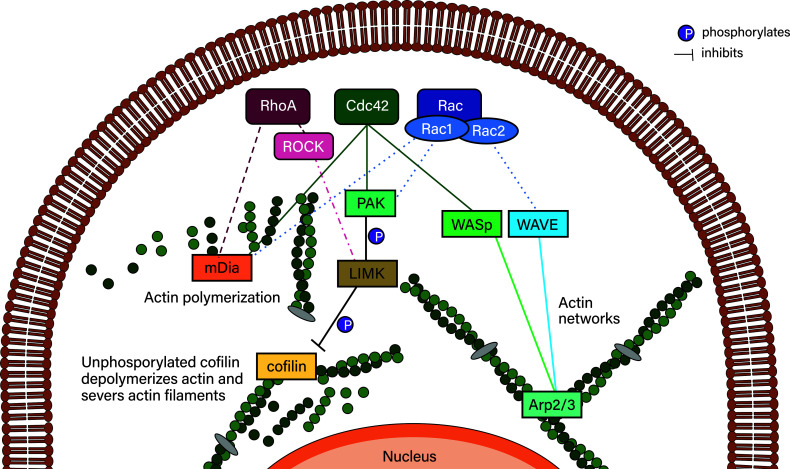
Simplified RhoGTPase signaling to their downstream effectors. RhoA signals to mDia2 and, *via* ROCK, can inhibit actin depolymerization *via* LIMK/cofilin. Cdc42 can activate Rac proteins, which activates PAK proteins that phosphorylate LIMK family proteins, which phosphorylate and thereby deactivate cofilin. Deactivated phosphorylated cofilin is reactivated by dephosphorylation mediated by slingshot proteins such as SSHL1 (not shown). Cdc42 also initiates actin polymerization *via* mDia2 and actin network formation (typically in lamellipodia/lamella) *via* WASp, which activates Arp2/3. Arp2/3 is also activated by the WAVE complex of which hem1 is an associated protein. WAVE is activated by Rac proteins such as Rac1 and Rac2. Rac proteins also activate mDia2 as well as the PAK/LIMK/cofilin axis.

Actin is indispensable for both migration modes. In accordance with the importance of the actin network, RhoGTPases – integral to actin remodeling – have been shown to be involved in many migrating leukocytes. RhoGTPases include 20 members and are excellently reviewed in (141). Of these 20 members, RhoA, Rac proteins and Cdc42 will be characterized below and their downstream effectors are depicted in **Figure 6**.

### RhoA

RhoA has been linked to cell migration in general. It has been shown to be essential for, for example, colon cancer (142), stellate cell (143) and mature osteoclast migration (144). In leukocytes, RhoA is important for actin contractility; this enables retraction of the cell rear during monocyte transmigration (145) and pushes cell migration in several cell types (68, 71, 73, 146), which is characteristic of amoeboid migration and its associated round cell shapes and weak adhesion (18, 71). However, RhoA plays a role in both active migration modes. RhoA induces ROCK, which regulates actin *via* LIMK and cofilin (141). This has been shown to be essential for leukocyte migration, especially in 2D conditions in which RhoA/ROCK is essential for establishing one leading membrane lamella and thereby directionality (147). RhoA also enhances the activity of mammalian Diaphanous-related formins (mDia)2, which is important for actin polymerization (148, 149).

RhoA also regulates leukocyte arrest on the endothelium, by mediating CD11a/CD18 conformational change into the active conformation as part of an inside-out signaling pathway (4, 150, 151) downstream of RAP1 (in thymocytes) (152). It is important to note that (β2)-integrins are not the only adhesion receptors upstream of RhoA. For example, P2Y_1_, a platelet purinergic receptor, has been shown to be upstream of RhoA activation, and both were essential for platelet-dependent leukocyte chemotaxis *in vitro* and leukocyte recruitment in thrombocytopenic mice (153).

### Rac Proteins

Rac, or Rac proteins (Rac1, Rac2, Rac3 and RhoG), is another group of GTPases that can activate RhoA in fibroblasts (154, 155). Rac proteins are critical for generating actin rich, stimulated lamellipodia formation and membrane ruffling, and are important for establishing adhesion sites to the extracellular matrix together with Cdc42 in macrophages (156). While Rac1 is ubiquitously (141) and RhoG widely expressed (157), Rac2 is mostly only expressed in cells of the hematopoietic lineage (158, 159), and Rac3 is mostly found in the brain (141, 160, 161). Rac, along with increased adhesion, is associated with increased spreading and the slower mesenchymal migration mode (68, 71, 73, 146). Rac in leukocytes is critically dependent on DOCK2, a haematopoietic cell-specific CDM family protein downstream of chemokine receptors (162). T-lymphocytes and B-lymphocytes deficient in DOCK2 exhibit homing and migratory defects *in vivo*, however, these migratory defects where not present in monocytes (162). Rac signals *via* several different downstream proteins to initiate lamellipodia formation. Actin polymerization is initiated by Rac *via* the WAVE complex, which activates actin-related protein (Arp) 2/3 that activates actin polymerization in a branched network (163), or Rac initiates mDia2 which mediates actin polymerization directly. Similarly to Cdc42, Rac initiates actin turnover *via* active P-21–activated kinases (PAK), LIM kinase (LIMK) and Cofilin (141).

### Cdc42

Cdc42 in turn can activate Rac1 (154, 155, 164). Not only does Cdc42 regulate the actin cytoskeleton, it also has an important function in regulating cell polarity, which is well conserved in many eukaryotic organisms (141). Cdc42 controls actin polymerization *via* WASp and IRSp53, which activate ARP2/3 and mDia2 – which initiates actin polymerization directly – and PAK, which inhibits actin turnover *via* LIMK and Cofilin (141).

### RhoGTPase Expression Profiles Differ Across Leukocytes

RhoGTPases are clearly involved in regulating adhesion and migration of many different leukocytes. However, expression levels differ across leukocyte subpopulations and even within monocytes. A study by van Helden et al. (165) investigated mRNA levels of RhoGTPases in neutrophils, differentiating dendritic cells and macrophages. They found that neutrophils expressed high levels of Cdc42, RhoQ, Rac1, Rac2, RhoA and RhoC and low and specific expression of RhoG, RhoB, RhoF and RhoV. While this GTPase expression profile was similar between different cell types, differences emerged during differentiation. During dendritic cell differentiation, Rac1 was up- and Rac2 downregulated and the dendritic cell populations expressed very low levels of RhoV, which was also present at low levels in macrophages and at higher levels in CD34+ progenitors. Macrophage GTPase expression profiles looked similar to those of dendritic cells, however, M-CSF–differentiated macrophages also expressed low levels of RhoBTB1. Apart from these minuscule differences, neutrophils, dendritic cells and macrophages differed regarding whether RhoA, Rac1, Rac2 or CdC42 was most expressed. The individual percentages were also dependent on which growth factor had been used to differentiate the cells. For example, GM-CSF–differentiated macrophages expressed high Rac2 levels while M-CSF–differentiated macrophages expressed high levels of RhoA.

Furthermore, RhoH is a GTPase only expressed in the haematopoietic linage (166). While myeloid cells and lymphocytes have both been shown to express RhoH, lymphocytes express high levels of this GTPase (167, 168). Thymocyte development and T-cell receptor signaling is critically dependent on RhoH (169, 170). B-cell receptor signaling is intact in RhoH-deficient cells, however B-cell chronic lymphocytic leukemia is dependent on RhoH (171). RhoH has been described as an inhibitor of other RhoGTPase activity, because it negatively regulates RhoA-induced NfKB and p38 signaling in Jurkat cells (172), however, its active contribution to T-cell receptor signaling does not involve Rac inhibition (170). Knockdown of RhoH in T-cells leads to the activation of CD11a/CD18, but does not affect the heterodimer’s ability to bind ligands and mediate adhesion (170). It is important to note that RhoH is regulated on a transcriptional level (172).

These results might explain some of the discrepancies between cell types and even studies. RhoGTPase expression profiles are clearly closely linked to individual cell identity, based on which stimuli the cell had experienced. Additionally, leukocyte migration mechanisms should be more closely investigated based on cell types and even sub-populations.

## β2-Integrins Regulate Nuclear Elements and Gene Expression in Leukocytes

Integrins are mechanosensors and they transmit mechanical forces directly, *via* the actin cytoskeleton, to the nucleus (173) and the nuclear lamina, consisting of lamin. Lamin A/C has been shown to be force-sensitive and scales in response to higher extracellular stiffness in order to maintain the cell’s tensional integrity (174, 175). Lamin A/C is responsible for nuclear stiffness (176) and high lamin A/C levels hinder 3D leukocyte migration through porous environments (92). If the β2-integrin/kindlin-3 binding is abolished by mutating the β2-cytoplasmic tail, lamin A/C levels are downregulated. Furthermore, this leads to an increase in H3K4me3 levels, a methylation mark associated with active transcription, changes in open chromatin formation and activation of an IKAROS network, which includes RelA. Compared to WT dendritic cells, dendritic cells that had an abolished β2-integrin/kindlin-3 interaction have been shown to migrate faster in a 3D collagen matrix, express higher levels of maturation surface receptors and induce superior tumor rejection responses in a B16.Ova and B16.F10 melanoma model. Directly targeting H3K4 methylation and disrupting the adhesion in WT dendritic cells was shown to be enough to replicate this phenotype (177). Confinement of Jurkat cells in a 3D collagen I matrix resulted in H3K4me3 upregulation *via* WD repeat domain 5 (178). Furthermore, in Jurkat cells or primary CD4+ T-cells VCAM1 adhesion mediated by α1β1-integrin leads to triple methylation of another lysine, H3K9me2/3. This involves interaction of methyltransferase G9a with lamin B1 and mediates nuclear stiffness and viscoelasticity and ultimately regulates T-cell migration in 2D and 3D as G9a depletion blocks T-cell migration (179). Finally, histone deacetylation has been shown to be essential for CD4+ T-cell extravasation as histone deacetylase 1 deficiency resulted in reduced transwell transmigration, aberrant migration on ICAM-1 surfaces *in vitro* and decreased migration into the intestinal epithelium and lamina propria *in vivo*. Additionally, during the cell adhesion cascade fewer histone deacetylase 1 deficient T-cells were found to probe the surface for places to transmigrate. Abolished histone deacetylation further resulted in downregulation of CD11a, CD18 and β7 chain as well as selectins on a transcriptional level, however, deficient T-cells spread more on ICAM-1 and formed more F-actin, while their random cell migration speed was increased by 10% (180). It is clear, that as with RhoGTPases, the mechanism of how epigenetic marks regulate leukocyte migration is complex and context dependent and much effort is required to unravel this mechanism fully.

## Characteristics of Different Leukocyte Migration

Leukocyte migration and specific migratory elements such as chemotaxis, transmigration, membrane architecture and adhesion are regulated differently by varying RhoGTPases and their downstream effectors across leukocyte cell types. In accordance with this, migratory behavior also differs between leukocyte cell types, however, some leukocytes have been much more extensively studied, while leukocytes such as basophils and eosinophils have not been studied much regarding migration and trafficking.

### Monocytes

Blood-derived monocytes enter tissues from the bloodstream *via* the cell adhesion cascade, which involves utilizing the 2D mesenchymal migration mode. Additionally, they are able to use the amoeboid migration mode in fibrous, porous collagen I matrices, however, they are unable to infiltrate dense matrigel matrices or to form podosomes (181). Successful invasion of such matrices is dependent on proteolytic degradation (see section on macrophages). Monocyte migration seems to be intracellularly governed by RhoA and ROCK, while Filamin A merely restricts monocyte transmigration *in vitro* (182). RhoA and ROCK control β2-integrin localization (145) and leading edge formation during transmigration (147). Furthermore, RhoA/ROCK promote monocyte adhesion, migration and transendothelial transmigration (183). RhoA, as well as Cdc42, regulate filopodia formation (184, 185), and Cdc42 also regulates monocyte migration across endothelial cells (185).

### Dendritic Cells

Dendritic cell migration is dependent on their maturity, with immature dendritic cells being relatively immobile, while sampling antigens and intrinsically activated or extrinsically stimulated maturation leads to CCR7 upregulation, which initiates haptotaxis (directed migration along an immobilized chemokine gradient) (186). Dendritic cells use different migration modes when migrating to lymph nodes. To exit constrained porous environments, such as in ear tissues and the skin, dendritic cells use the amoeboid migration mode (18, 187, 188). Like macrophages, dendritic cells can use a protease-dependent, ROCK-independent migration mode in dense 3D environments like matrigel. In such dense 3D environments, podosome formation, rather than maturation status, has been shown to be essential for 3D migration. Dendritic cells migrate through heterogenous environments such as tumor spheroids using both mesenchymal and amoeboid migration modes (188). Once dendritic cells are inside the lymph vessels, they form lamellipodia to crawl along the endothelium (55). CCL-21/CCR7 activation initiates chemotaxis/haptotaxis (189, 190) and arrests dendritic cells on the endothelium. In addition to the chemokine cue, dendritic cells also sense the lymph flow which directs them to proceed downstream (55). Dendritic cells can enter lymph nodes by transmigrating through the subcapsular sinus on the afferent side and then prepare the entry of T-cells into these sites. During intranodal migration, dendritic cells have been shown to exhibit extensive polarization, which established leading edges and a long uropod, which was necessary to translocate to the paracortex. Dendritic cell polarization is dependent on CCR7 (191). Motile dendritic cells are also found in the gut, the lamina propria, Peyer’s patches and solitary intestinal lymphoid tissues from where dendritic cells migrate to gut-draining lymph nodes. During gut infections accompanied by rupture of afferent lymph vessels, dendritic cell migration is prevented (186). Dendritic cell migration from the lung tissue to draining lymph nodes has been shown to be dependent on CCR7, but CCR8 was also implicated (186, 192). Intracellularly, β2-integrin/kindlin-3 signaling restricts the mature, migratory phenotype of dendritic cells, and abolishing the β2-integrin/kindlin-3 interaction leads to increased expression of maturity markers, increased migration speed in 3D towards CCL19, increased IL-12a production and induction of superior anti-tumor responses in B16.OVA and B16.F10 melanoma models *in vivo* (177, 193). Furthermore, in dendritic cells the β2-integrin/kindlin-3 signaling is upstream of RhoA in a ROCK-independent pathway that regulates adhesion but not 3D migration (17).

### Macrophages

Macrophages use mesenchymal migration modes, as well as amoeboid migration modes in 3D environments, and this depends on the specific environment/matrix architecture. For example, the amoeboid migration mode is utilized in porous fibrillar collagen I, and mesenchymal migration mode in dense matrigel (194). During mesenchymal migration modes, macrophages form podosomes that can lyse collagen, however, this migration mode is not dependent on matrix metalloproteinase (MMPs) and might be mediated by other proteolytic systems (194). If collagen I is in the form of a gel, macrophages also utilize mesenchymal migration modes and this could be due to the increased stiffness and viscosity of this form of collagen I (194) or the matrix structure, such as the availability of pores. Macrophages use a migration mode dependent on ROCK and MMPs to infiltrate tumor cell spheroids encapsulated in matrigel (195). These spheroids would present a heterogeneous matrix architecture similar to another collagen I matrix polymerized at 3.6 mg/ml. The 3.6 mg/ml collagen I matrix has been found to contain fibrillar collagen structures, be porous and have intermediate stiffness (G’ is around 30 Pa, though this is quite soft) and viscosity (G’’ is around 8–9 Pa) (195). Macrophages used a combination of amoeboid migration mode and MMP-dependent mesenchymal migration mode in this collagen I matrix, which resembled spheroid infiltration. In a follow-up study, Filamin A was shown to be essential for a macrophage mesenchymal migration mode utilizing proteolytic podosomes. Filamin A is, however, dispensable for amoeboid migration modes (196), and restricts macrophage transmigration *in vitro* (197). Macrophage migration mode seems to be additionally dependent on macrophage status and β2-integrin expression levels (198). CD11d/CD18 is upregulated on M1 proinflammatory macrophages, while CD11b/CD18 is highly expressed on resident macrophages. High expression levels of these integrins inhibit migration and promote adhesion, while intermediate integrin expression promotes amoeboid and mesenchymal migration modes. Knocking out these heterodimers has also been shown to result in reduced migration (198). Finally, individual levels of RhoGTPases seem to be critical for macrophage migration: RhoA mediates actin cable formation, macrophage rounding up and contractility (156) and has to be precisely concentrated in order to facilitate macrophage migration (199). Rac1 and Rac2 are important GTPases in macrophages (200), with Rac1 being the most abundant Rac isoform (201, 202) and being essential for lamellipodia formation and macrophage migration (199). Rac1 is also important for macrophage invasion through matrigel (203), which would have likely utilized the mesenchymal migration mode (194). Rac2 mediates transmigration; additionally, its role in macrophage migration is somewhat substratum-dependent, since it mediates migration across laminin and fibronectin (203).

### Microglia

Microglia are a type of leukocyte found in the brain. They express CD11b (a β2-integrin alpha chain) (204, 205) and are phenotypically similar to macrophages. While a study with β2-integrin knockout mice has shown that β2-integrins are not necessary for microglia migration (206), similar to dendritic cells, this experiment reduced β2-integrins to just 2–16 % of CD18 expression (206), therefore not precluding their involvement to a small degree. Microglia *in vitro* were shown to migrate with similar speeds (ca. 60 µm/min) to leukocytes. During migration, microglia were shown to use thin transparent lamellipodia as ‘sails’ to glide forward (207). In murine brain slices, microglia migrated at an average of 5.02 ± 0.06 μm/min, with spurts of 10 μm/min speeds and migrated individually and not highly directionally (208). In another study using murine brain slices, CD11a was knocked down in a microglia cell line and this inhibited microglia migration. Although CD11a-deficient microglia were able to migrate up to 50 µm into the slices, WT microglia were able to migrate down to 80 µm (209).

### Neutrophils

Neutrophils are capable of using the mesenchymal migration mode, as well as the amoeboid migration mode (18). In matrices such as fibrillar, porous collagen I matrices, neutrophils use the amoeboid migration mode, however, they are unable to invade dense matrigel matrices or to form podosomes (181). It is important to note that neutrophils express adhesion receptors dynamically (210); for example, α4β1-integrin expression is downregulated during neutrophil maturation (14). Neutrophils have been observed to swarm into tissues and form clusters as a response to sterile inflammation or infections with bacteria, fungi and parasites. Neutrophil swarming behavior can be categorized into two patterns, transient swarms and persistent swarms. Transient swarms are formed by neutrophils migrating towards a chemoattractant, forming a cluster for a few minutes and migrating out again to join nearby clusters. Persistent swarms are formed by sustained neutrophil migration into clusters that can last for hours. There seems to be tissue-specific parameters dictating which kind of swarming behavior occurs, although in some tissues swarming behaviors can also co-occur. Transient swarms occur in lungs, lymph nodes and intestine, while persistent swarms occur in skin, liver, spleen and cornea. Further parameters that have been shown to influence which swarming behavior occurs are the extent of initial and secondary tissue damage, presence of pathogens and number of recruited neutrophils (211). CD11a/CD18 and CD11b/CD18 were both shown to be essential for the final stages of accumulation in collagen-free zones (212).

The role of β2-integrin cofactors in neutrophil trafficking is dependent on the dimensionality of the environment: in 2D talin mediates adhesion, spreading, polarization and migration, while in 3D it plays a role in chemotactic velocity (213, 214). Furthermore, talin is important for activating integrins to mediate neutrophil entry into sites of infections, to arrest in venules and to emigrate out of venules (214). Kindlin-3 mediates directionality in neutrophils crawling on 2D surfaces (213). Filamin A seems to be an important regulator of neutrophil migration. Not only does it enable fast migration speed in 2D and 3D (215), it also mediates RhoA activation and localization in uropods. Furthermore, Filamin A regulates myosin II–mediated uropod contractility *via* RhoA, and Filamin A mediates spreading on β1- and β2-integrin ligands (216, 217).

Different RhoGTPases regulate different aspects of neutrophil migration: Rac1 is important for directed neutrophil migration (218), while Rac2 mediates chemokinesis (random movement), spreading as well as proper F-actin generation, which regulates polarization and directed movement (219, 220). Conversely, Cdc42 restricts neutrophil migration speed, but is important for proper directed migration and steering *in vitro* (221, 222). Cdc42 regulates directed neutrophil migration by controlling polarity *via* its effector Wiskott-Aldrich Syndrome Protein (WASp), whose activation and localization in uropods is controlled by Cdc42. WASp in turn controls CD11b localization in uropods, and the integrin heterodimer then recruits and stabilizes tubulin (223).

### Eosinophils and Basophils

Eosinophil transmigration has been mainly shown to be mediated by CD11a/CD18 and CD11b/CD18 when the cells were stimulated by IL-1. IL-4–mediated transmigration, however, seems to be dependent on α4β1 (VLA-4). Inhibiting CD11b/CD18 and α4β1 has been shown to impair eosinophil accumulation in the airways in *in vivo* models (10). Similarly, inhibiting CD18 with antibodies inhibited basophil transendothelial transmigration in transwell assays (224).

### Mast Cells

Mast cell progenitors traffic from the bone marrow into tissues where they mature, and their homing to different (healthy and inflamed mucosal) tissues is dependent on different integrins (225, 226). Homing to the intestine is dependent on integrin α4β7 (the α4 chain, which can also pair with β1, but especially the β7 chain in this case) (21, 225), while homing to the lungs is dependent on α4β7 as well as α4β1 (20, 225). Mast cell homing to the peritoneum is dependent on CD11b/CD18 and αIIbβ3 (23, 227). Migrating mast cells form pericentral actin clusters that prevent cell flattening and localize secretory granules at the cell center, and this actin organization is mDia(1) dependent. mDia1 knockdown was shown to impair mast cell transmigration in a transwell model, however, mDia1 knockout mice have normal mast cell distribution in tissues (228). Mast cells migrate in response to UV exposure from the skin to draining lymph nodes, and this is important for immune suppression. The extent of such migrating mast cells is dependent on the degree of UV exposure, and mast cells primarily migrate to B-cell areas in lymph nodes guided by CXCL12 secreted by B-cells (receptor CXCR4) (54). Additionally, mast cells accumulate at sites of angiogenesis (i.e. blood vessel formation), and are guided by angiogenic factors, such as VEGF, PDGF-AB and bFGF (229). Interestingly, mast cells can secrete heparin, a polysaccharide, and use it to induce endothelial cell migration *in vitro* (230).

### NK Cells

Similarly to T-cells and B-cells, NK cells exit the bone marrow to enter secondary lymphoid organs in a β2-integrin–dependent mode (231). In lymph nodes, NK cells are activated by dendritic cells during infections, however, *in vivo* they have been shown to barely form stable dendritic cell interactions, instead forming short-lived contacts with dendritic cells. During this process, NK cells are highly motile in order to establish multiple interactions with dendritic cells and migrate with a less straight trajectory than T-cells (232). Conversely, in superficial lymph node regions, NK cells have been shown to exhibit low motility and long interactions with dendritic cells (233). On stroma, human NK cell subsets have different migratory profiles, with CD56 ^bright^ NK cells migrating in short, multidirectional tracks, CD56 ^dim^ NK cells migrating in linear and rapid fashion and CD56 ^neg^ NK cells (CD56^low^ CD3^−^ CD16^+^ CD57^+^ KIR^+^) migrating to a minimal extent. NK cell migration is dependent on CD56 expression, and more mature NK cells exhibit the most motility (234). Furthermore, NK cell migration on 2D surfaces *in vitro* can consist of a series of short, constrained motions mixed with highly directed motions or migration in a straight line for the entirety of migration, the latter being observed in few NK cells (235). NK cells are proposed to be capable of transmigration and invasion in response to chemokine and cytokine stimulation and without being activated by dendritic cells. NK cell invasion into cancer spheroids was further proposed to be β2-integrin–dependent and, unlike T-cells, NK cells do not recirculate (236).

### T-Cells

T-cells in the afferent lymph system are transported by lymph flow into the medullary sinus system. These T-cells then cross the endothelium and enter lymph nodes by directly transmigrating into medullary cords (191). Intranodal migration of T-cells has been shown to be dependent on CCR7, with the chemokine receptor stimulating migration in T-cell areas but not in subcapsular regions *in vivo* (237). T-cells exit the lymph nodes and travel to sites of infections, which involves adhesion to the endothelium under shear conditions. As mentioned, this adhesion is dependent on the CD18/Kindlin-3 interaction (238). Arrested T-cells that crawl on endothelial cells and ICAM-1–coated surfaces form lamellipodia, which contain low amounts of CD11a/CD18, and uropods, which contain high amounts of CD11a/CD18. Additionally, T-cells that crawl on ICAM-1, incorporated in lipid bilayers, form a specific zone in the middle of their bodies. This zone contains high affinity CD11a/CD18 and mediates adhesion to and migration on ICAM-1, and its stability is dependent on talin (239). In tissues as well as fibrillar, porous collagen I matrixes, T-cells – like monocytes and neutrophils – can use the amoeboid migration mode (181). However T-cells are unable to form podosomes or to invade dense matrigel matrices (181). Intracellularly, T-cell homing and trafficking is critically dependent on Kindlin-3, which mediates CD11a/CD18 inside-out signaling (125, 238). The involvement of further β2-integrin signaling components for T-cell trafficking is context dependent. T-cell transmigration is dependent on RhoA (240), while the tight binding of Filamin A to integrin tails inhibits transmigration (134). Rac1 signaling mediates T-cell adhesion (241) and can, together with Cdc42, compensate for loss of β2-integrin/14-3-3 signaling to mediate T-cell spreading (108).

### B-Cells

B-cells are capable of using the amoeboid migration mode (242, 243), however, their 2D migration seems somewhat atypical. During migration on CXCL13-coated surfaces, the leading membrane edge has been shown to undergo dilation and shrinking events, and the balance of these events was predictive of turning versus directional persistence. During this migration, an imbalance of dilation versus shrinking events was associated with changes in direction (244). This phenomenon might involve β2-integrins, since CXCL13/CXCR5 mediates CD11a/CD18 adhesion in B-cells (243). In large B-cell lymphoma, the amoeboid migration mode is mediated by an IL-10-JAK-STAT-3-RhoH pathway that was shown to increase RhoA activity and suppress tubulin acetylation (245).

### Leukocyte Migration in Tumors

Leukocyte migration also has a huge implication in cancer therapy, as therapeutic antibodies targeting immune checkpoints have revolutionized cancer treatment in recent years. However, for 70-80% of patients their tumors do not respond to this treatment (246). This poor response has been linked to the presence or absence of T-cells in the tumor environments (247). T-cell tumor invasion is hindered by the prolonged interaction of T-cells with tumor-associated macrophages (TAMs), and depletion of TAMs can restore T-cell migration and invasion (248). In order to mount an anti-tumor immune response, antigen-presenting cells have to present antigens to and fully activate T-cells first. However, this mechanism is warped in order to support tumor growth by dendritic cell–like cells in the tumor periphery that engage CD8+ T-cells in long interactions without fully activating the T-cells (249, 250). This results in ‘immune excluded’ tumors with leukocytes stuck in the periphery. Macrophages and dendritic cells have, in turn, been found to be regulated by myeloid-derived suppressor cells (MDSCs) in glioma (251). MDSCs arise as monocytes from the bone marrow and then effectuate homing to secondary lymphatic organs before being recruited into the tumor, where they are retained. This process is thought to be a result of crosstalk between the bone marrow and the tumor, involving growth factors and chemokines produced in the tumor microenvironment. MDSC homing culminates in their retention in the tumor microenvironment, however, less is known about this process then about T-cell retention (252). Our understanding of leukocyte migration is limited in general and is even more elusive in regards to how it is impaired on a molecular level specifically in tumors.

## Conclusion

It is now established in the literature that leukocytes use a distinct migration mode – the amoeboid migration mode – which sets them apart from other migrating cells. However, there are distinct differences between migration profiles of leukocyte populations, and especially the individual molecular mechanisms that mediate these, which were broadly outlined in this review. Future research will hopefully flesh out these differences and should address exactly how the molecular balance between RhoGTPases is regulated. The focus of this review was on β2-integrins – some of the most studied adhesion receptors. However, there are many other adhesion and surface receptors that signal *via* RhoGTPases and have been shown to play a role in leukocyte migration, such as NOTCH (253–255) and cadherins (256–258). Incorporating other ECM ligands for these receptors could reveal more RhoGTPase profiles and even new migration modes or unique leukocyte migration behavior. Furthermore, individual surface receptors might be key regulators or could strengthen and perpetuate signaling. The ultimate goal would be to establish exactly how collective surface receptor signaling mediates distinct RhoGTPase profiles and how this translates to exact migratory behavior. This should also include unraveling how leukocyte migration is regulated by histone methylation and acetylation downstream of integrin and adhesion receptor signaling. Thus far, deacetylation, H3K4me3, as well as H3K9me2/3, have been implicated in dendritic cell and T-cell migration, respectively. It would be important to see if other methylation marks are associated with the migration of distinct leukocyte subpopulations. Moreover, histone methylation and acetylation have received the most attention thus far but are only a small portion of the many types of histone modifications: it is possible that the others also play a role in regulating leukocyte migration.

Progress in cancer therapy has focused on combination treatments. Combining immune checkpoint inhibitors as well as combination therapy using radiotherapy first and then immune checkpoint inhibitors can improve objective response rates as well as progression free survival (259, 260). Ultimately similar improvements could be possible by mapping out the intracellular signaling that governs leukocyte migration. This could empower us to reestablish migratory behavior in effector T-cells stuck in the periphery of immune-excluded tumors, which could further increase patient survival rates after immune checkpoint inhibitor combination treatment, as well as control leukocyte migration in other diseases.

## Author Contributions

The author confirms being the sole contributor of this work and has approved it for publication.

## Funding

IFReC Kishimoto Foundation for supporting the study with a fellowship.

## Conflict of Interest

The author declares that the research was conducted in the absence of any commercial or financial relationships that could be construed as a potential conflict of interest.

## Publisher’s Note

All claims expressed in this article are solely those of the authors and do not necessarily represent those of their affiliated organizations, or those of the publisher, the editors and the reviewers. Any product that may be evaluated in this article, or claim that may be made by its manufacturer, is not guaranteed or endorsed by the publisher.
